# Exploring the molecular mechanism of notoginsenoside R1 in sepsis-induced cardiomyopathy based on network pharmacology and experiments validation

**DOI:** 10.3389/fphar.2023.1101240

**Published:** 2023-01-13

**Authors:** Ruifei Shao, Wei Li, Rui Chen, Kunlin Li, Yu Cao, Guobing Chen, Lihong Jiang

**Affiliations:** ^1^ Faculty of Life Science and Technology, Kunming University of Science and Technology, Kunming, China; ^2^ Medical School, Kunming University of Science and Technology, Kunming, China; ^3^ Yan’an Hospital Affiliated to Kunming Medical University, Kunming, China; ^4^ Department of Cardiovascular Surgery, The First Peoples’ Hospital of Yunnan Province, Kunming, China; ^5^ Department of Emergency Medicine, The First People’s Hospital of Yunnan Province, Kunming, China; ^6^ Yunnan Key Laboratory of Innovative Application of Traditional Chinese Medicine, Department of Cardiovascular Surgery, The First People’s Hospital of Yunnan Province, The Affiliated Hospital of Kunming University of Science and Technology, Kunming, China

**Keywords:** notoginsenoside R1, sepsis-induced cardiomyopathy, network pharmacology, molecular docking, tumor necrosis factor-alpha

## Abstract

Sepsis-induced cardiomyopathy (SIC) is an important manifestation of sepsis, and abnormal cardiac function affects the development of sepsis. Notoginsenoside R1 (NG-R1) is a unique bioactive component of Panax notoginseng with anti-inflammatory and antioxidant effects. However, the effects and possible mechanisms of NG-R1 on SIC are not clear. The purpose of this study was to identify the potential targets and regulatory mechanisms of the action of NG-R1 on SIC. To investigate the potential mechanism, we used network pharmacology, molecular docking, qRT-PCR, and immunofluorescence. The results showed that NG-R1 ameliorated myocardial fibrosis in septic mice. Validation of network pharmacology and molecular docking results revealed that NG-R1 reduced tumor necrosis factor-Alpha (TNF-α) expression in myocardial tissues and AC16 cardiomyocytes in mice, as well as inflammatory factor release in AC16 cells, so TNF-α may be a potential target of NG-R1 against SIC. The present study demonstrated that NG-R1 could protect against SIC and by regulating the expression of TNF-α inflammatory factors, providing a new idea for sepsis drug development.

## 1 Introduction

Sepsis is now defined as life-threatening organ dysfunction caused by a unbalanced host response to infection (Singer et al., 2016). Notably, the new emphasis on organ dysfunction rather than infection stems from a growing understanding of sepsis pathophysiology, which includes both inflammatory and anti-inflammatory responses (Jacob, 2016). As the dominant cause of high morbidity and mortality in hospitalized patients, sepsis is characterized by excessively released inflammatory mediators and frequently leads to multiple organ failure impacting most commonly of the cardiovascular and respiratory systems, and septic cardiomyopathy contributes to this outcome (Hershey and Kahn, 2017; Rhee et al., 2017; Cecconi et al., 2018). As an important manifestation of sepsis, sepsis-induced cardiomyopathy (SIC) is characterized by left ventricular dilatation and depressed ejection fraction, and eventually progressed quickly to circulatory compromise, microcirculatory alterations and mitochondrial damage in cardiomyocytes (Hollenberg and Singer, 2021; Xie et al., 2021). Sepsis patient mortality is directly related to systolic and diastolic cardiac dysfunction, indicating that the prognosis of critically ill sepsis patients is dependent on global cardiac dysfunction. Because cardiac function returns to normal after 2 weeks of sepsis, functional rather than structural cardiac abnormalities have been suggested (Landesberg et al., 2012; Martin et al., 2019).

Panax notoginseng (Burk.) F. H. Chen (also known as Sanqi) has been used for thousands of years in China to promote blood circulation, remove blood stasis, induce hemostasis, and relieve swelling and pain (Huo et al., 2022). Notoginsenoside R1 belongs to the protopanax triol type and is the main monomeric component isolated from Panax ginseng that exerts anti-inflammatory, antioxidant, and anti-apoptotic effects (Pang et al., 2017). Studies have shown that NG-R1 can inhibit the inflammatory response during sepsis through multiple targets and pathways. NG-R1 has direct anti-inflammatory and anti-apoptotic effects on cardiomyocytes and can improve septic cardiac dysfunction and attenuate the inflammatory response in mice, but its mechanism is not clear (Jiang et al., 2015; Zhong et al., 2015).

Network pharmacology integrates bioinformatics, systems biology, and polypharmacology to reveal the complex biological network relationships between drugs, genes, targets, and diseases. Network pharmacology emphasizes multi-pathway regulation *via* signaling pathways, and on this basis analyzes and predicts drug pharmacological mechanisms, verifies and evaluates drug efficacy, adverse effects, and mechanisms of action *via* corresponding experiments, discovers highly efficient and less toxic drugs to improve drug therapeutic effects and reduce toxic side effects, and thus improves the success rate of new drug clinical trials (Sakle et al., 2020; Gouthami et al., 2022). Molecular docking is a drug design method based on receptor characteristics and the mode of interaction between receptors and drug molecules. It is a theoretical simulation method that primarily studies intermolecular interactions (e.g., ligand and receptor) and predicts binding modes and affinity (Hopkins, 2008).

**FIGURE 1:**
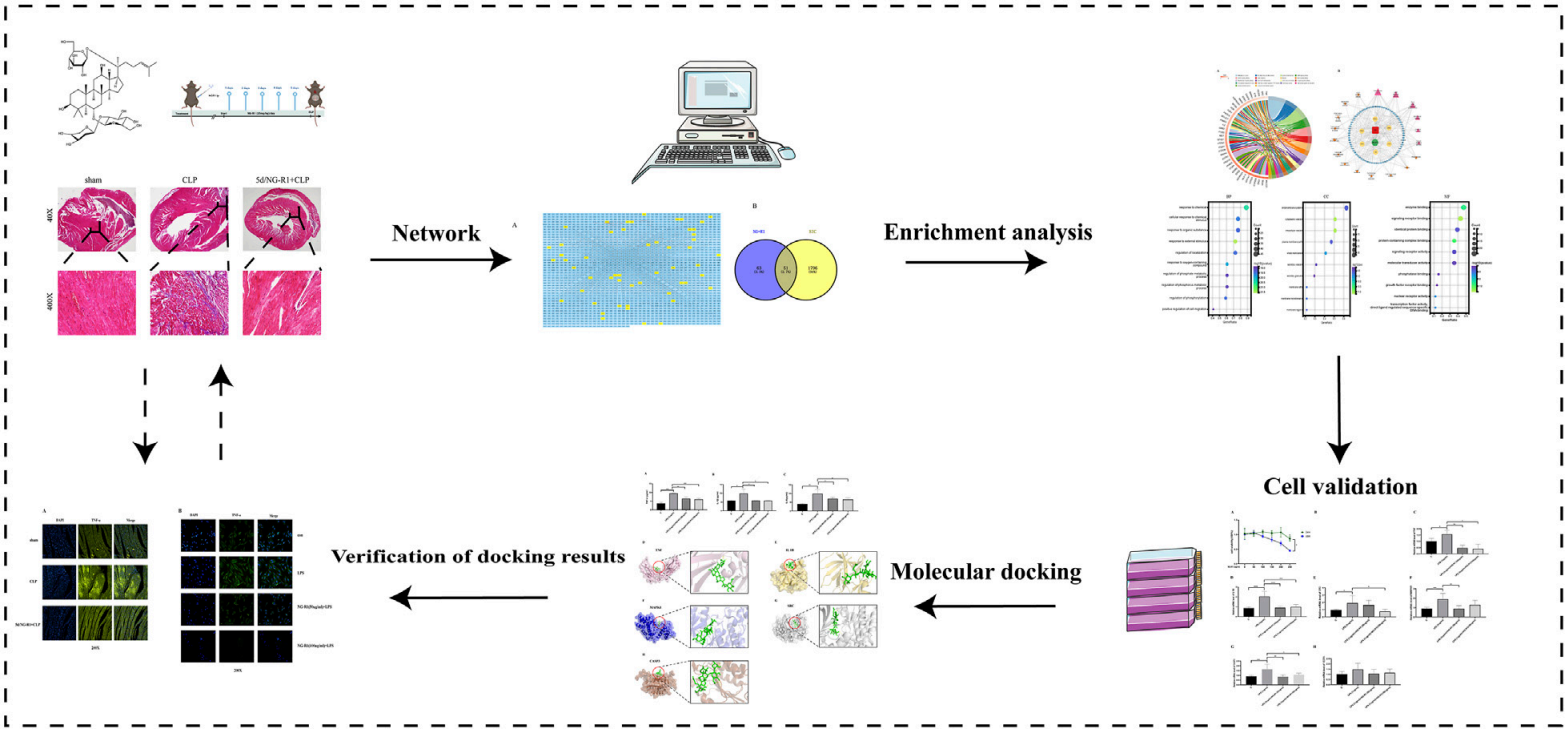
The detailed flow chart of the study.

This study combines a network pharmacology approach with molecular docking techniques and experimental validation to further explore the role and mechanism of NG-R1 in SIC. This research will shed new light on the search for effective drugs to treat SIC. Figure 1 shows the flow chart for this study.

## 2 Materials and methods

### 2.1 Screening of NG-R1 drug targets and SIC disease targets

The potential targets of notoginsenoside R1 were screened through Swiss Target Prediction (http://www.swisstargetprediction.ch/) (Daina et al., 2019), Encyclopedia of Traditional Chinese Medicine (http://www.tcmip.cn/ETCM/index.php/Home/Index/All) (Xu et al., 2019). The molecular targets involved in the pathological progression of sepsis cardiomyopathy were screened through GeneCards (https://www.genecards.org/), pharmgkb (https://www.pharmgkb.org/) (Lee et al., 2022) and OMIM(https://omim.org/) databases using “sepsis-induced cardiomyopathy” as keywords and followed by filtering with the term “*Homo sapiens*”, and the repetitive targets were removed. Targets obtained by NG-R1 and SIC were summarized, overlapped and executed in Microsoft Excel and plotted in Venny 2.1 (bioinfogp.cnb.csic.es/tools/Venny/) to confirm the targets shared by the NGR1-SIC.

### 2.2 PPI network of compound-disease targets

Protein-Protein Interaction Networks (PPI) are used to explore the interactions of proteins with each other and their mutual functional connections, and the STRING11.0 database (https://string-db.org) is commonly used to construct protein interaction networks for common target genes of drugs and diseases (Szklarczyk et al., 2017). In the database we entered NGR1-SIC common targets, selected the minimum interaction threshold as “medium confidence>0.4″, the organism was set as “Homosapiens”, and the unconnected nodes in the network were hidden. In addition, we built a PPI network using Cytoscape 3.9.1 (Otasek et al., 2019) to visualize the PPI network as a result based on the contribution (degree) value of the target. The larger the degree value of this node, the darker the shape and color.

### 2.3 Hub gene analysis

The hub gene is usually a gene with high degree value, which is obtained by computer algorithm with high connectivity degree in the gene expression network. After analyzing the degree values of each target in the PPI network through the cytohubba plugin in Cytoscape software, the core targets were ranked according to the degree values and betweenness centrality (the larger the betweenness parameters, the more important the target is in the network; the filtering criterion was more than twice the median of the degree values).

### 2.4 GO enrichment and KEGG pathway analysis

Gene Ontolog (GO) database is to qualify and describe the functions of genes and proteins, while Kyoto Encyclopedia of Genes and Genomes (KEGG) enrichment analysis is to integrate these protein functions to computationally find the relevant signaling pathways. We submitted the key targets shared by NGR1-SIC in the network to Metascape (https://metascape.org/) (Zhou et al., 2019) for GO and KEGG pathway enrichment analysis. The *p*-value for enrichment was determined, and differences were deemed statistically significant at *p* < 0.05. Finally, Cytoscape 3.9.1 was used to build a compound-target-pathway network based on the outcomes of the KEGG investigation.

### 2.5 Molecular docking

The 2D structure of NG-R1 (small molecule ligand) was first retrieved from PubChem (https://pubchem.ncbi.nlm.nih.gov/#query=Notoginsenoside%20R1) (Kim et al., 2021) and exported in SDF file mode, and the 2D structure was converted to 3D using ChemDraw and exported as MOL2, small molecule compounds were imported into AutoDock Tools-1.5.7 software (Martin et al., 2021) and atomic charges were added, atom types were defaulted, all elastic bonds were rotatable, and then the files were finally saved in pdbqt format for preservation. Next, the crystal structures of key targets (macromolecular receptors) were obtained using the RCSB Protein Data Bank (https://www.rcsb.org/) (Burley et al., 2021). Pymol 1.7 software was then applied to remove excess small molecules and the AutoDock Tools1.5.7 program was used to enter the proteins, remove water molecules, add hydrogen atoms, and save them as pdbqt folders. Finally, AutoDock was used to perform bulk molecular docking, Pymol 1.7 was used to visualize NG-R1 with the protein, and finally the component-target with the lower affinity score was selected for further investigation.

### 2.6 Experimental assessment

#### 2.6.1 Reagents and chemicals

Notoginsenoside R1 (pure: ≥98%) (B21099) was purchased from Shanghai Yuanye (Shanghai, China). RIPA lysis buffer (R0010) and BCA Kit (PC0010) were purchased from Solarbio (China). Masson trichrome staining Kit (G1346) was purchased from Solarbio (China). The primary antibody TNF-α (Proteintech, China), CoraLite 488 Conjugated-Goat anti rabbit IgG (Proteintech, China).

#### 2.6.2 Animals and treatment

Male C57BL/6 mice weighing approximately 22 ± 2 g (provided by the Department of Zoology, Kunming Medical University) were raised in pathogen-free cages at the Experimental Animal Center of Kunming Medical University in a controlled temperature (22°C ± 1°C) environment with relative humidity (45%–55%). The mice were housed under a light/dark cycle, and water and food were available *adlibitum* during the experiment.

Construction of CLP (cecum ligation perforation, CLP) sepsis model: Before surgery, surgical instruments were autoclaved and mice were anesthetized with sodium pentobarbital (50 mg/kg). Under aseptic conditions, a 1.5 cm incision was made in the lower abdomen to expose the cecum. The distal end of the cecum was completely ligated with 3.0 silk from the end 1 cm, punctured once with an 18-gauge needle, and then replaced in the peritoneal cavity. The peritoneal wall and skin were then closed with double sutures. After surgery, the mice were resuscitated intraperitoneally with 1 ml of sterile saline (0.9%) fluid. Mice in the sham group had the abdominal incision and cecum exposed without ligation or puncture. After surgery, mice had access to water and food.

Experimental grouping: After 1 week of adaptive feeding, mice were randomly divided into three groups: Sham group (sham-operated group), CLP group (sepsis group), and 5d/NG-R1+CLP group (sepsis group after NG-R1 interference). Mice were injected intraperitoneally with 25 mg/kg NG-R1 once a day for 5 days. Mice in the sham and CLP groups were injected intraperitoneally with 200 ul saline once a day for 5 days. All mice were constructed as CLP sepsis models on day 6, and relevant samples were collected by execution 24 h after surgery for subsequent molecular biology experiments.

This study was conducted according to the National Institutes of Health guidelines and approved by the Research Ethics Committee of Kunming Medical University (No. Kmmu20221605).

#### 2.6.3 Masson’s trichrome staining

After the mice were anesthetized with sodium pentobarbital (50 mg/kg), the skin was cut from the submandibular by continuing upward from the raphe, and the skin and subcutaneous tissue were bluntly peeled off to both sides to expose the thorax and superficial cervical musculature. The thorax and pleura were cut along the median to expose the heart, find the aortic arch, clip the aorta, cut the artery, remove the heart, and rinse with saline. Fresh myocardial tissue was fixed in 4% paraformaldehyde, embedded in paraffin, and cut into serial slices of 5 μm thickness. Staining was performed using masson staining kits.

#### 2.6.4 Cell culture and treatment with drugs

AC16 cells were maintained in Dulbecco’s modified Eagle medium F12 (DMEM-F12) supplemented with 10% fetalbovine serum (FBS) and 1% penicillin–streptomycin at 37°C in a humidified atmosphere of 95% air and 5% CO2. AC16 cells were seeded in six-well culture plate at a density of 2 × 10^5^ per well. LPS is dissolved insterile water, filtered and stored at −20°C in aliquots and protected from light. After 24 h, the cells were harvested for the following experiments. The cells were co-incubated with LPS (1ug/ml) and NG-R1 (50 ug/ml, 100 ug/ml) for 24 h and then harvested for the assay.

#### 2.6.5 The cell viability assay

AC16 cells were incubated with LPS (1ug/ml)/LPS (1ug/ml)+different concentration-dependent NG-R1 for 24 or 48 h, or different concentration-dependent NG-R1 alone for 24 h or 48 h. Cell viability was measured using the CCK-8 Assay Kit (Dojindo, Kumamoto, Japan) according to the protocol. The results were obtained from three independent experiments, and each experiment was performed in triplicate.

#### 2.6.6 Quantitative real-time polymerase chain reaction (qRT-PCR)

Total RNA was extracted using the TRIZOL method (Invitrogen, Carlsbad, CA). The mRNA was reversetranscribed into cDNA and SYBR Green PCR Master Mix (Thermo Fisher Scientific, Waltham, MA) was used to determine the transcriptional expression of specific genes. Finally, the prepared cDNA was placed on the Roche Real Time PCR Plate and quantified in the PCR system. The primer synthesis of the target gene is as Table 1. Relative geneexpression was calculated using the 2^−ΔΔCT^ method.

**TABLE 1:** Primer sequence of target gene.

Primer	Forward	Reverse
H-TNF	5′-GTT​CCT​CAG​CCT​CTT​CTC​CT	5′-ACA​ACA​TGG​GCT​ACA​GGC​TT-3′
H-SRC	5′-GAG​CGG​CTC​CAG​ATT​GTC​AA-3′	5′-CTG​GGG​ATG​TAG​CCT​GTC​TGT-3′
H-VEGFA	5′-GTG​TCC​AGT​GTA​GAT​GAA-3′	5′-CCT​GTT​CTC​TGT​TAT​GTT​G-3′
H-MAPK1	5′-CAG​TTC​TTG​ACC​CCT​GGT​CC-3′	5′-TAC​ATA​CTG​CCG​CAG​GTC​AC-3′
H-IL1B	5′-ATG​ATG​GCT​TAT​TAC​AGT​GGC​AA-3′	5′-GTC​GGA​GAT​TCG​TAG​CTG​GA-3′
H-CASP3	5′-CAT​GGA​AGC​GAA​TCA​ATG​GAC​T-3′	5′-CTG​TAC​CAG​ACC​GAG​ATG​TCA-3′
H-GAPDH	5′-CGA​CCA​CTT​TGT​CAA​GCT​CA-3	5′-AGGGGTCTACA GGCAACTG-3′

#### 2.6.7 Enzyme linked immunosorbent assay (ELISA)

Mice peripheral blood: All mice were anesthetized with sodium pentobarbital (50 mg/kg) and blood was collected *via* orbital vein, centrifuged at 800 g (3,000 rpm) for 30 min at 4°C, and serum was separated for enzyme-linked immunosorbent assay detection. Serum samples were incubated with the corresponding antibodies for 30 min at 37°C in incubation wells, followed by five washes of 30 s each with concentrated wash buffer. The reaction was then terminated by adding a color developer and incubating at 37°C for 10 min followed by the addition of a termination solution. Finally, the optical density (OD) were measured at 450 nm using a Thermo Scientific microplate reader. The mouse CK-MB and cTnT ELISA kits were purchased from Cusabio Biotech Co. (Wu Han, China).

Cell culture supernatant: After the completion of modeling, the cell culture supernatant was collected and centrifuged at 1000 g (3,750 rpm) for 20 min at 4°C, and the supernatant was used for enzyme-linked immunosorbent assay detection. The supernatant was incubated with horseradish peroxidase (HRP)-labeled antibody in incubation wells for 60 min at 37°C, followed by five washes of 1 min each with concentrated wash buffer. The reaction was subsequently terminated by adding the chromogenic agent and incubating at 37°C for 15 min followed by the addition of the termination solution. Finally, the optical density (OD) were measured at 450 nm using a Thermo Scientific microplate reader. The Human TNF-α, IL-1β and IL-6 ELISA kits were purchased from Mlbio Co., Ltd. (Shanghai, China).

#### 2.6.8 Immunofluorescence analysis

Cellular immunofluorescence: AC16 cells after modeling, remove the culture medium, wash it with PBST for 3 times, and then fix it with 4% paraformaldehyde for 4 h. After that, remove paraformaldehyde, wash it with PBS for 3 times, then break the membrane with 1% Triton X-100 for 10 min, remove Triton X-100, wash it with PBST for 3 times, and then sealed it with 5% BSA for 2 h. Finally, an appropriate amount of primary antibody TNF-α (1:200 dilution, Proteintech, China) was added and incubated at 4°C overnight. The primary antibody was washed in PBS for 3 × 10 min, then mixed with CoraLite 488 Conjugated-Goat anti rabbit IgG (1:500 dilution, Proteintech, China) was added and incubated at room temperature in the dark for 2 h. PBS was used to wash secondary antibody for 3 × 10 min, and 4,6-diamidino-2-phenylindole (DAPI, Sigma, USA) was used to stain the cell nuclei and block the slices. The nuclei were observed and photographed using a fuorescence microscope (Olympus, Japan).

Tissue surface fluorescence: Fresh myocardial tissues were embedded in paraffin, fixed in 4% paraformaldehyde, and cut into serial sections 5 μm thickness. The sections were dewaxed and washed three times with PBS before being incubated for 20 min with an appropriate amount of closed endogenous peroxidase (methanol:30% hydrogen peroxide 9:1) and washed three times with PBS. The tissue was then drilled for 10 min with 0.25% Triton X-100 and rinsed three times with PBS. After being closed with 5% goat serum for 2 h at room temperature, PBS was used to prepare primary antibodies at appropriate concentrations. Incubate for 2 h at room temperature before transferring to 4°C overnight. The next day, PBS was rinsed three times before incubating CoraLite 488 Conjugated-Goat anti rabbit IgG for 2 hours. After washing, the tissues were incubated for 1 h with 0.1% DAPI before being examined under a fluorescence microscope.

### 2.7 Statistical analysis

Data were expressed as mean ± SD values. Statistical analysis was performed by using SPSS software. The one-way ANOVA followed by a *post hoc* Bonferroni multiple comparison test was used to compare control and treated groups. *p*-value less than 0.05 was considered statistically significant. All blots are representative of experiments that were performed at least three times.

## 3 Results

### 3.1 NG-R1 increased the survival rate and alleviated myocardial injury in sepsis mice

**FIGURE 2:**
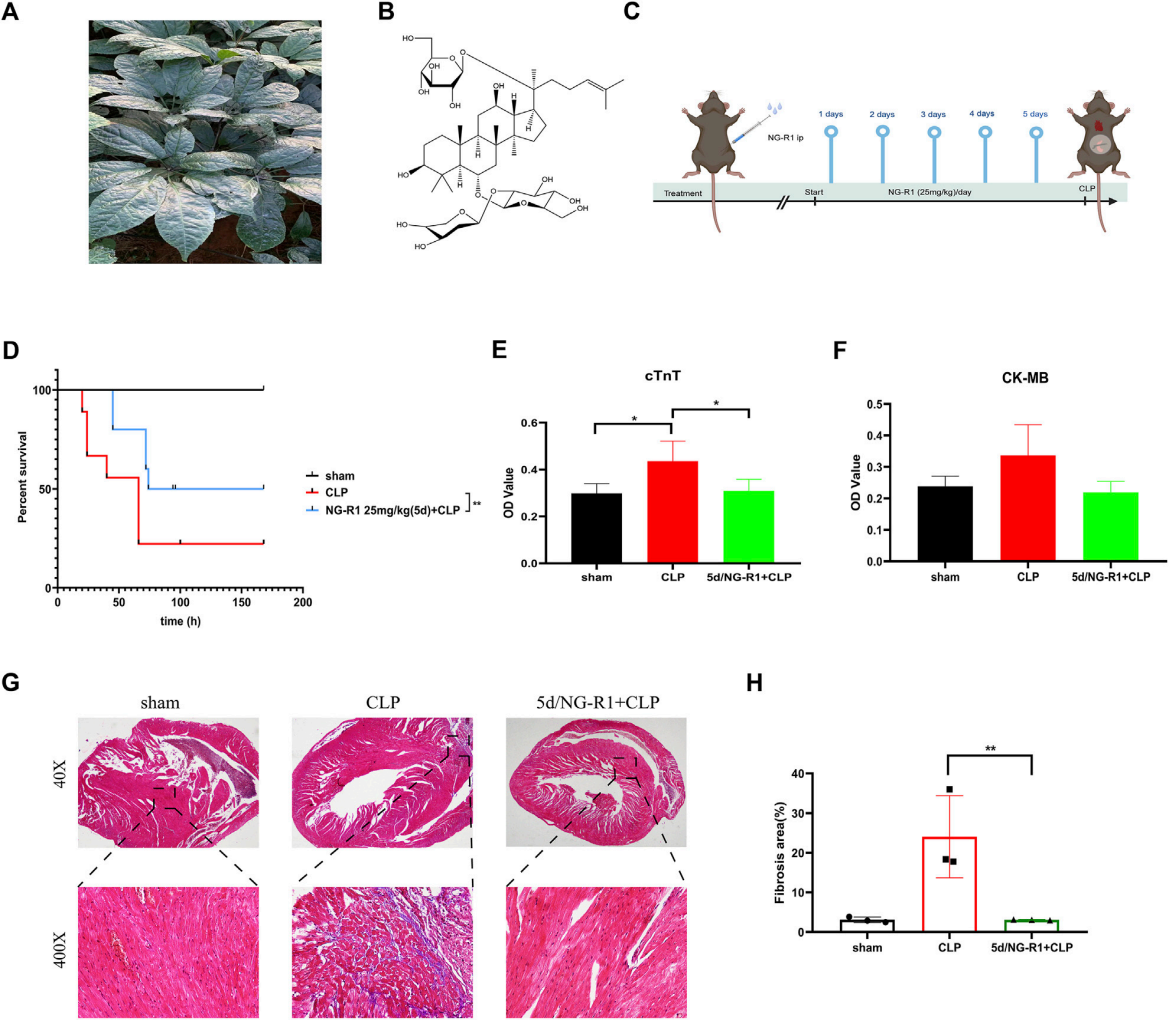
NG-R1 increased the survival rate and alleviated myocardial injury in sepsis mice. **(A, B)** Schematic diagram of Panax ginseng plant, chemical structure of Notoginsenoside R1, **(C)** Flow chart of animal experiments. **(D)** 7-day survival rates of mice between different subgroups after NG-R1 intervention (n = 10 per group). **(E, F)** OD values of cTnT, CK-MB in peripheral blood of mice (OD values are proportional to expression) (*n* = 4 per group). **(G, H)** Statistics of myocardial tissue masson stainingin different groups (40X) (400X), fibrosis area (*n* = 3 per group). The data are represented as means ± SD; *p < 0.05, **p < 0.01.

NG-R1 exerts anti-inflammatory and antioxidant effects in various diseases (Gong et al., 2022; Zhang et al., 2022). However, the mechanism of action of NG-R1 in sepsis-induced myocardial injury is unclear. Therefore, we pretreated Male C57BL/6 mice with NG-R1 intraperitoneally for 5 days before constructing the CLP model as the NG-R1 intervention group (Figure 2C). Postoperatively, we observed the 7 days survival rate of mice in each group, and the results showed that the 7 days survival rate was 100% in the sham group, 70% in the CLP group after 24 h and 20% in the CLP group after 7 days. Interestingly, the survival rate of animals in the 5d/NG-R1 group was significantly higher, with 24 h and 7 days survival rates of 90% and 50%, respectively, while the mice in the 5d/NG-R1 group had better activity and mental status compared with animals in the CLP group (Figure 2D).

Next, we examined the expression of cTnT (cardiac troponin T, cTnT) and CK-MB (creatine kinase-MB isoform, CK-MB) as markers of myocardial injury in the peripheral blood of mice. The results showed a statistically significant increase in cTnT expression in peripheral blood after CLP intervention and a significant decrease after NG-R1 intervention. In contrast, there was a trend of increased but not statistically significant expression of CK-MB and a trend of decreased expression after NG-R1 intervention (Figures 2E,F). Meanwhile, the results of masson staining showed that the myocardial tissue of sham group mice had scattered distribution or striated collagen fibers and intact myocardial fibers; while the myocardial fibers in the CLP group were broken in the center of the myocardial tissue, with thickened collagen fibers and increased deposition; surprisingly, the degree of myocardial fibrosis in the NG-R1 pretreated CLP group was moderated and approached that of the sham group (Figures 2G,H). This suggests that NG-R1 has a protective effect on sepsis-induced myocardial injury, but the mechanism is unclear.

### 3.2 NGR1-SIC target screening and network analysis

**FIGURE 3:**
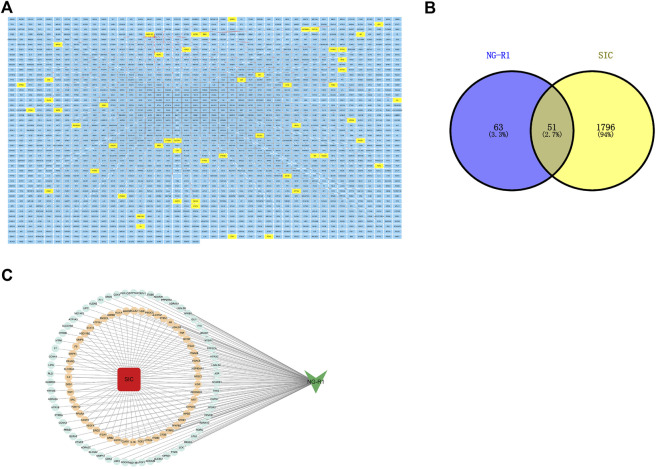
Target screening. **(A)** NG-R1 and SIC target summary, with NGR1-SIC common targets highlighted in yellow. **(B)** Venn analysis of NG-R1 and SIC crossover targets. **(C)** Data visualization of NG-R1 and SIC-related targets.

The molecular formula of NG-R1 is C_47_H_80_O_18,_ molecular weight 933.1 (Figures 2A, B). We found the corresponding targets of NG-R1 in STP and ETCM databases, removed duplicate genes, and finally obtained 114 corresponding targets. We found 1847 corresponding targets of SIC in GeneCards, pharmgkb, OMIM databases (Figure 3A). According to the Venn diagram of NG-R1 intersecting with SIC targets, there were 51 overlapping targets (Figures 3B,C) (Supplementary Table S1). These results suggest that 45% of NG-R1 targets are associated with sepsis myocardial injury.

### 3.3 PPI network construction

**FIGURE 4:**
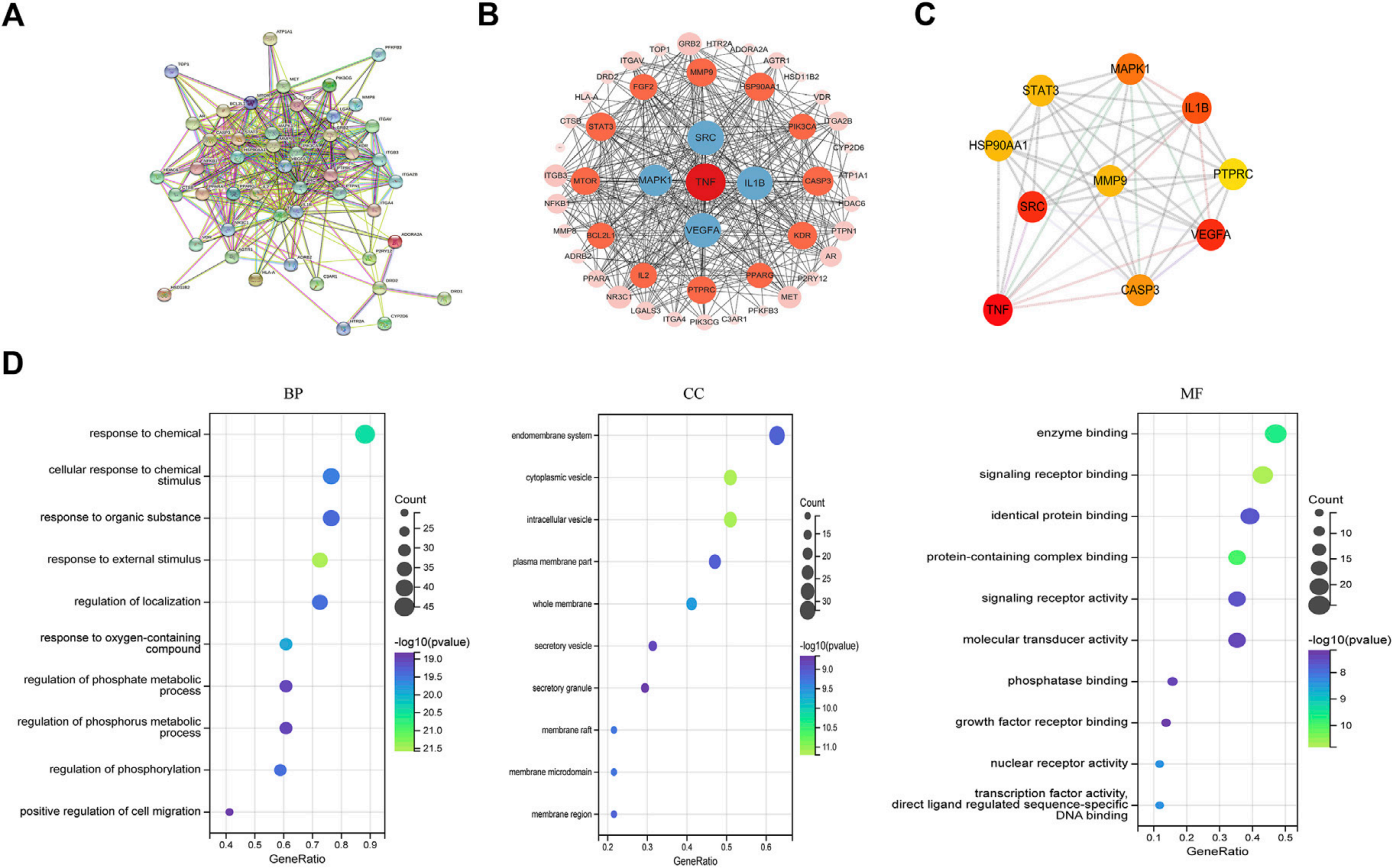
Screening for core targets and analysis of NGR1-SIC PPI protein interactions. **(A, B)** Network of NGR1-SIC related target PPI interaction. **(C)** Screening of the top ten core targets from a list of 59. **(D)** The 51 targets’ GO enrichment analysis.

The PPI network between NG-R1 and SIC was constructed and the key targets were searched. We input the 51 common targets obtained from the analysis into the String database for analysis, hide disconnected nodes in the network, and obtain the PPI network graph (Figure 4A). The interaction network has 48 nodes, 377 edges, and an average degree of 14.8. The PPI network was imported into Cytoscape 3.9.1 software for analysis, and the most important indicators associated with NGR1-SIC were obtained, with larger areas and darker colors indicating more critical indicators (Figure 4B). Topological analysis was performed using Cytohubba plug-in, and the top 10 core genes with high degree values were obtained as TNF, SRC, VEGFA, IL1B, MAPK1, CASP3, HSP90AA1, STAT3, MMP9 and PTPRC, and the clustering analysis table shows the detailed results (Figure 4C) (Table 2).

**TABLE 2:** Designations and topological parameters of hub components in the PPI network.

NO.	Gene	Degree	Betweenness centrality
1	TNF	38	238.42137
2	SRC	34	189.21949
3	VEGFA	34	142.48912
4	IL1B	33	174.94983
5	MAPK1	30	70.24041
6	CASP3	27	49.73996
7	HSP90AA1	26	45.4178
8	STAT3	26	28.13809
9	MMP9	26	35.74681
10	PTPRC	25	66.00984

### 3.4 Go and KEGG analysis

The 48 targets of common significance for drugs and diseases were subjected to GO and KEGG pathway analysis.

The top ten most important GO categories are shown in Figure 4D. Among the biological processes (BPs), 927 BPs were enriched, and the targets were mainly enriched in positive regulation of response to chemical, response to organic substance, regulation of localization, regulation of phosphorylation. In cellular component (CC), 69 CCs were enriched, targets were mainly enriched in endomembrane system, cytoplasmic vesicle, intracellular vesicle, plasma membrane part. In addition, a total of 66 MFs were enriched in the molecular function (MF) class, with targets mainly enriched in enzyme binding, signaling receptor binding, identical protein binding, protein-containing complex binding, signaling receptor activity. The enrichment analysis of core targets provides direction for future experimental validation.

**FIGURE 5:**
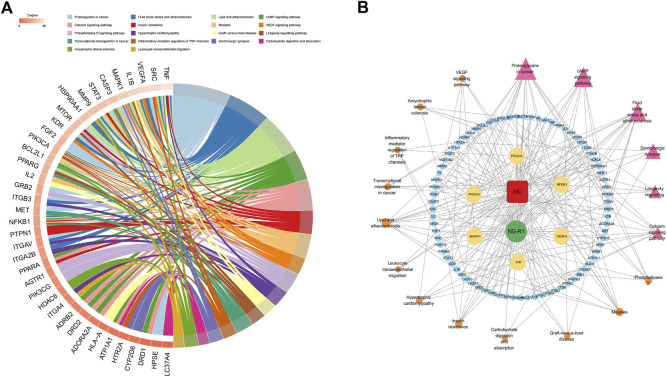
KEGG pathway and target-pathway network diagram construction. **(A)** The 51 targets’ KEGG enrichment analysis. **(B)** Multidimensional network diagram of the first 18 KEGG pathways’ common targets and information.

KEGG analysis was performed on the targets shared by NG-R1 and SIC, and a total of 150 pathways were obtained. A pathway-target network was constructed for the top 18 KEGG pathways that were mainly enriched for the targets (Figure 5A) (Table 3), and the main pathways were found to be enriched for Lipid and atherosclerosis (hsa05417), cAMP signaling pathway (hsa04024), VEGF signaling pathway (hsa04370), inflammatory mediator regulation of TRP channels (hsa04750), exhibiting the multi-target and multi-pathway characteristics of NG-R1 against septic myocardial injury. A large body of literature suggests that septic myocardial injury is associated with inflammation (Glenn and Goldman, 1976; Honda et al., 2019; Xie et al., 2021), and NG-R1 is highly involved in anti-inflammatory regulation (Jiao et al., 2021; Li et al., 2022). Therefore, we hypothesize that the potential mechanism of NG-R1 for sepsis-induced cardiomyopathy may be the regulation of inflammation-related pathways.

**TABLE 3:** KEGG analysis of NG-R1 therapy for SIC.

NO.	Pathway name	Log10(P)	Gene number
hsa05205	Proteoglycans in cancer	−22.18	16
hsa05418	Fluid shear stress and atherosclerosis	−17.12	12
hsa05417	Lipid and atherosclerosis	−14.81	12
hsa04024	cAMP signaling pathway	−9.92	9
hsa04020	Calcium signaling pathway	−9.61	9
hsa04931	Insulin resistance	−9.2	7
hsa05162	Measles	−8.43	7
hsa04370	VEGF signaling pathway	−7.28	5
hsa04072	Phospholipase D signaling pathway	−6.73	6
hsa05410	Hypertrophic cardiomyopathy	−6.35	5
hsa05332	Graft-versus-host disease	−6.11	4
hsa04211	Longevity regulating pathway	−4.8	4
hsa05202	Transcriptional misregulation in cancer	−4.73	5
hsa04750	Inflammatory mediator regulation of TRP channels	−4.63	4
hsa04726	Serotonergic synapse	−4.36	4
hsa04973	Carbohydrate digestion and absorption	−4.16	3
hsa05014	Amyotrophic lateral sclerosis	−3.43	5
hsa04670	Leukocyte transendothelial migration	−3.02	3

### 3.5 Drug-disease-pathway

The above drug, disease, key target and KEGG pathway information was used to construct a multidimensional network diagram and analyze the relationship between NG-R1-disease-key pathway. As obtained from the interactions between the nodes of the multidimensional network graph (Figure 5B), NG-R1 could improve myocardial injury caused by sepsis through multiple pathways and targets. The TNF inflammatory pathway may be an important signaling pathway for NG-R1 mediated SIC treatment.

### 3.6 Molecular docking

In this study, molecular docking analysis was used to determine whether the key targets had good stability with the corresponding active ingredients. Binding energy is considered to be one of the key indicators to verify the stability of the conformation of the bound protein and active ingredient (Sun et al., 2022). Molecular docking of hub genes involved in the inflammatory pathway and cAMP pathway to NG-R1 was performed using Autodock Vina software by the comprehensive network pharmacology analysis described above. The results showed meaningful negative docking energy of NG-R1 with TNF, SRC, IL1B, MAPK1 and CASP3, where NG-R1 formed four hydrogen bonds with TNF, one hydrogen bond with IL1B, three hydrogen bonds with MAPK1, five hydrogen bonds with SRC and five hydrogen bonds with CASP3 (Figures 7D–H). Binding energies were calculated to assess the degree of complementarity between small molecules and large proteins, and lower binding energies indicated higher stability (Fu et al., 2022). While the binding energies between TNF, SRC, IL1B, MAPK1, CASP3 and NG-R1 were −6.8 kcal/mol, −8.9 kcal/mol, −6.8 kcal/mol, −8.6 kcal/mol and −5.7 kcal/mol (Table 4). The above results are consistent with the results of our KEGG analysis.

**TABLE 4:** The binding energy of NG-R1 and core targets (kcal/mol).

Gene	Affinity (kcal/mol)	PDB ID
TNF	−6.8	6ugy
SRC	−8.9	7ng7
IL1B	−6.8	1twe
MAPK1	−8.6	4zzn
CASP3	−5.7	2j32

### 3.7 Experimental assessment

#### 3.7.1 AC16 cell viability is increased by NG-R1

**FIGURE 6:**
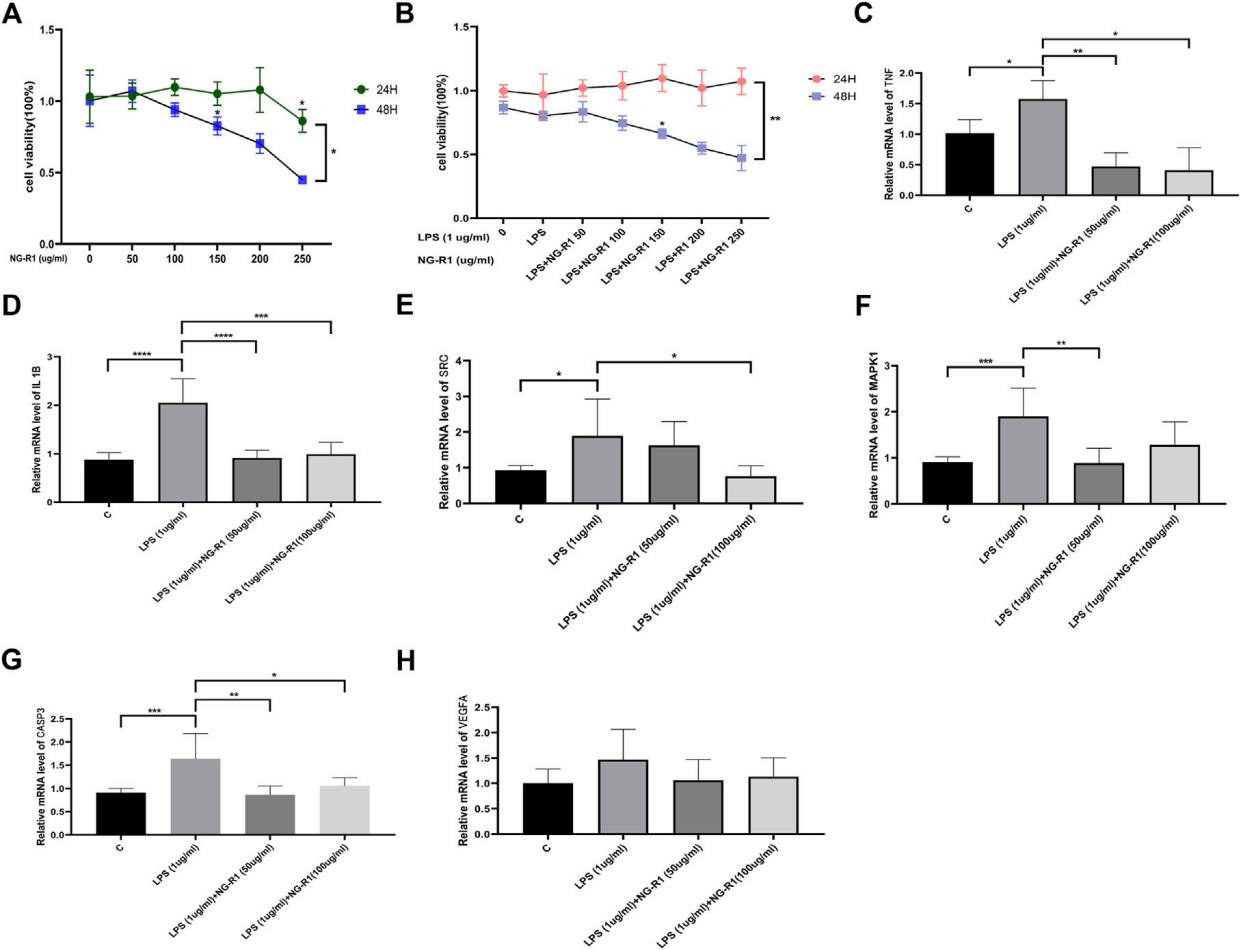
AC16 human cardiomyocytes were validated *in vitro*. **(A)** The effect of different NG-R1 concentrations (0, 50, 100, 150, 200, and 250 ug/ml) on the viability of AC16 cardiomyocytes over time (24 and 48 h), (*n* = 3 per group). **(B)** Effect of different concentrations of NG-R1 (0, 50, 100, 150, 200, 250 ug/ml) and LPS (1ug/ml) on the viability of AC16 cardiomyocytes after co-incubation for different times (24 h and 48 h) (*n* = 3 per group). **(C–H)** Analysis of changes in the expression of core genes TNF, IL1B, SRC, MAPK1 and CASP3 transcript levels after LPS, NG-R1 intervention (*n* = 3 per group). The data are represented as means ± SD; *p < 0.05, **p < 0.01, ***p < 0.001, ****p < 0.0001.

We designed *in vitro* experiments using a human cardiomyocyte cell line (AC16) to further validate the predicted results of network pharmacology of NG-R1 in septic myocardial injury. To begin, we used different NG-R1 concentrations (0, 50, 100, 150, 200, and 250 ug/ml) to stimulate AC16 cells for 24 or 48 h to determine the experimental concentration and time of NG-R1, and the results showed a concentration-dependent decrease in cell viability under 24 h 200 ug/ml NG-R1 and 48 h 100 ug/ml NG-R1 treatment (Figure 6A). Next, we used 1ug/ml LPS to stimulate AC16 cells to create a sepsis myocardial injury cell model, while co-incubating for 24 h or 48 h with the above-mentioned different concentrations of NG-R1, and the results showed that 24 h NG-R1 increased the viability of LPS-induced AC16 cells, but at 48 h 100 ug/ml NG-R1 treatment showed a concentration-dependent decrease in cell viability (Figure 6B). As a result, for modeling, we chose 50, 100 ug/ml NG-R1 with 1ug/ml LPS co-incubated for 24 h.

#### 3.7.2 NGR1-SIC screening core target validation

Based on the network pharmacology core target data, we examined the expression of TNF, MAPK1, SRC, IL1B, CASP3, and VEGFA transcript levels in AC16 cells co-incubated with 50 ug/ml, 100 ug/ml NG-R1 and 1ug/ml LPS for 24 h. TNF, MAPK1, SRC, IL1B, and CASP3 transcript levels were significantly reduced after co-incubation with NG-R1 compared to the LPS stimulation group (Figures 6C–G), but VEGFA transcript levels were not significantly different (Figure 6H).

#### 3.7.3 NG-R1 reduces LPS-induced inflammatory expression in AC16 cells

**FIGURE 7:**
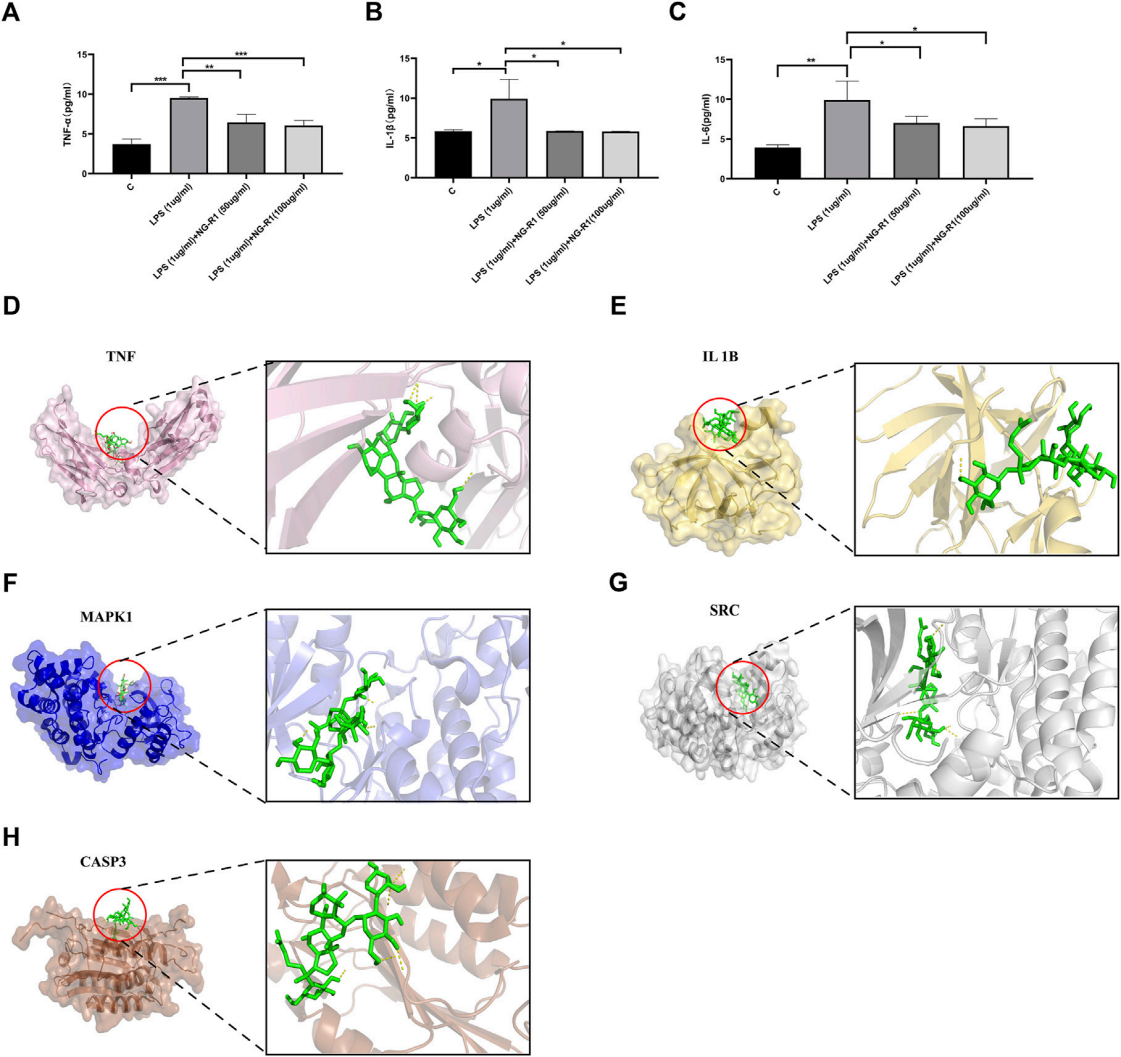
Analysis of inflammatory factor expression and molecular docking results in myocardial injury cells after NG-R1 treatment. **(A–C)** ELISA assay results show the levels of TNF-α, IL-1β and IL-6 in AC16 cardiomyocytes of different treatment groups (*n* = 3 per group). **(D)** Docking results of TNF and NG-R1 with docking energy −6.8 kcal/mol (surface structure and cartoon structure). **(E)** Docking results for IL1B and NG-R1, docking energy −6.8 kcal/mol (surface structure and cartoon structure). **(F)** Docking results of MAPK1 and NG-R1 with docking energy −8.6 kcal/mol (surface structure and cartoon structure). **(G)** Docking results of SRC and NG-R1 with docking energy −8.9 kcal/mol (surface structure and cartoon structure). **(H)** Docking results of CASP3 and NG-R1 with docking energy -5.7 kcal/mol (surface structure and cartoon structure). The data are represented as means ± SD; *p < 0.05, **p < 0.01, ***p < 0.001.

The KEGG enrichment pathway and molecular docking results indicated that inflammatory signaling pathways may be involved in the development of NGR1-SIC. Therefore, we examined the expression of inflammatory factors in the supernatants of AC16 cells co-incubated for 24 h with 50, 100 ug/ml NG-R1, and 1ug/ml LPS. The results showed that the expression of TNF-α, IL-6, and IL-1β were significantly increased after LPS stimulation, while their expression was significantly decreased after NG-R1 intervention (Figures 7A–C). This suggests that NG-R1 reduces the inflammatory response in sepsis-induced myocardial injury.

#### 3.7.4 NG-R1 reduced TNF-α expression in AC16 cells and myocardial tissue

**FIGURE 8:**
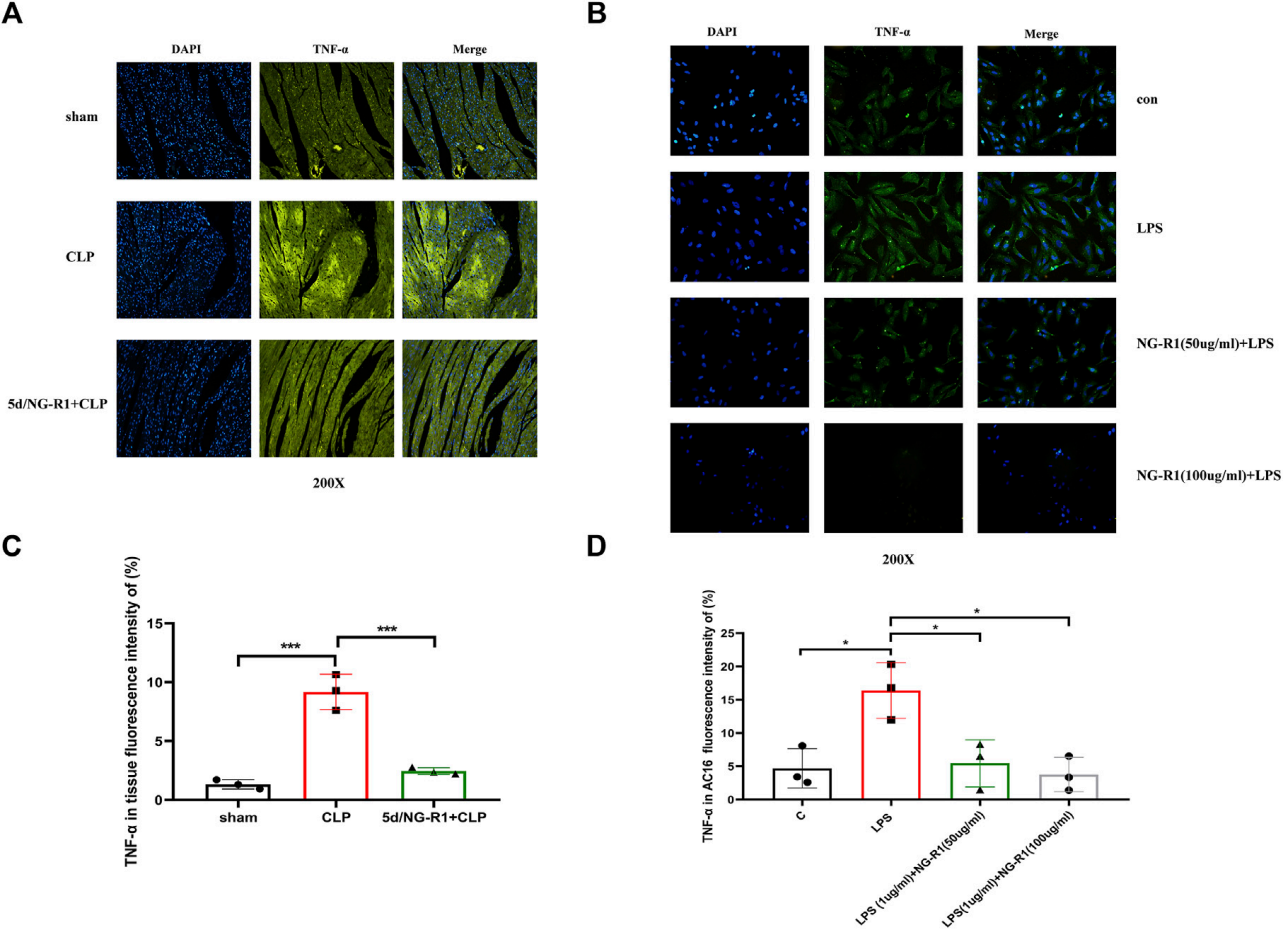
NG-R1 reduces TNF-α expression in myocardial tissues and AC16 cardiomyocytes. **(A, C)** TNF-α immunofluorescence expression in mice tissues, fluorescence intensity statistics (n = 3 per group) (200X). **(B, D)** TNF-α immunofluorescence expression in AC16 cardiomyocytes, fluorescence intensity statistics (n = 3 per group) (200X). The data are represented as means ± SD; *p < 0.05, ***p < 0.001.

TNF-α is a cytokine with pleiotropic effects on various cell types and has been identified is a major regulator of the inflammatory response. NG-R1 was shown to alleviate rheumatoid arthritis in TNF-tg mice by inhibiting the TNF-α signaling pathway to promote lymphatic drainage function (Jiao et al., 2021). The results of our network pharmacology and molecular docking also indicated TNF-α as a key gene for NG-R1 and SIC, so we investigated the role of TNF-α in NGR1-SIC in combination with *in vitro* and *in vivo* experiments. We used NG-R1 to intervene in a sepsis animal model and an AC16 cell model, respectively (Figure 2C) (Figure 6B). TNF-α was then immunofluorescence localized in AC16 cells and mice myocardial tissues. The fluorescence intensity of TNF-α in myocardial tissues of mice in the CLP group was significantly higher than the control and NG-R1-treated groups, according to the findings (Figures 8A, C). Meanwhile, in AC16 cells, TNF-α fluorescence intensity was significantly higher in the LPS group than the control group, but significantly lower in the 50 ug/ml NG-R1 and 100 ug/ml NG-R1 intervention groups (Figures 8B, D).

## 4 Discussion

Mountainous evidence suggests that inflammation, cardiomyocyte apoptosis and pyroptosis are involved in the development of sepsis and sepsis-induced cardiomyopathy (SIC) (Honda et al., 2019; Li et al., 2019). However, the dysregulation of the intense inflammatory response caused by tissue damage contributes to excessive cardiac fibrosis, making the heart function abnormally (Paulus and Zile, 2021).

Panax notoginseng (Burk.) F.H. Chen is the dried root and rhizome of Panax notoginseng, which has the function of dispersing blood stasis and stopping bleeding, reducing swelling and pain (Wang et al., 2021). More experimental studies have confirmed the protective effects of Notoginseng R1 on the cardiovascular system, which can effectively reduce oxidative stress and inflammatory responses (Zhu et al., 2021; Li et al., 2022). However, the mechanism of action of NG-R1 in SIC is unclear, which aroused our interest. In recent years, network pharmacology has become one of the frontiers and hot spots in the field of TCM research (Yang et al., 2022). Therefore, we used a combination of network pharmacology and experimental validation to further investigate the mechanism of action of NG-R1 in SIC.

First, in the animal model, the survival rate of septic mice was significantly increased and the expression of CK-MB in peripheral blood was significantly decreased after NG-R1 intervention. We also observed cardiac fibrosis in the sepsis group of mice, whereas cardiac fibrosis was reduced in the NGR1 intervention group. Following that, we identified 51 NG-R1 and SIC common targets using network pharmacology and screened 10 key targets. Second, enrichment analysis of the 51 targets revealed that NG-R1 treatment of SIC was primarily related to cAMP and inflammatory signaling pathways. The results of molecular docking revealed that NG-R1 had a low docking score with TNF-α, indicating its good binding ability. Because NG-R1 was shown to alleviate rheumatoid arthritis in TNF-Tg mice by promoting lymphatic drainage function *via* inhibition of the TNF-α signaling pathway (Jiao et al., 2021), so we validated it in the inflammatory signaling pathway.

Second, we used AC16 human cardiomyocytes to create an *in vitro* model of myocardial injury in sepsis. First, we found that NG-R1 and LPS co-treatment increased cardiomyocyte viability. Second, we looked at the transcriptional levels of the first six core targets identified in network pharmacology and discovered that TNF, IL1B, MAPK1, SRC and CASP3 transcript levels were significantly lower in the NG-R1 and LPS co-treatment groups compared to the LPS group. We also looked at inflammatory factors and discovered that the expression of TNF-α, IL-6, and IL-1β in the cell supernatant was significantly lower after NGR1 treatment compared to the LPS group. Finally, combined with the results of molecular docking, we did immunofluorescence of TNF-α in AC16 cells and heart tissues after modeling, and found that the fluorescence intensity of TNF-α in AC16 cells after NG-R1 treatment was significantly reduced compared with the LPS group, and the fluorescence intensity of TNF-α in heart tissues after NGR1 treatment was also significantly reduced compared with the sepsis group.

**FIGURE 9:**
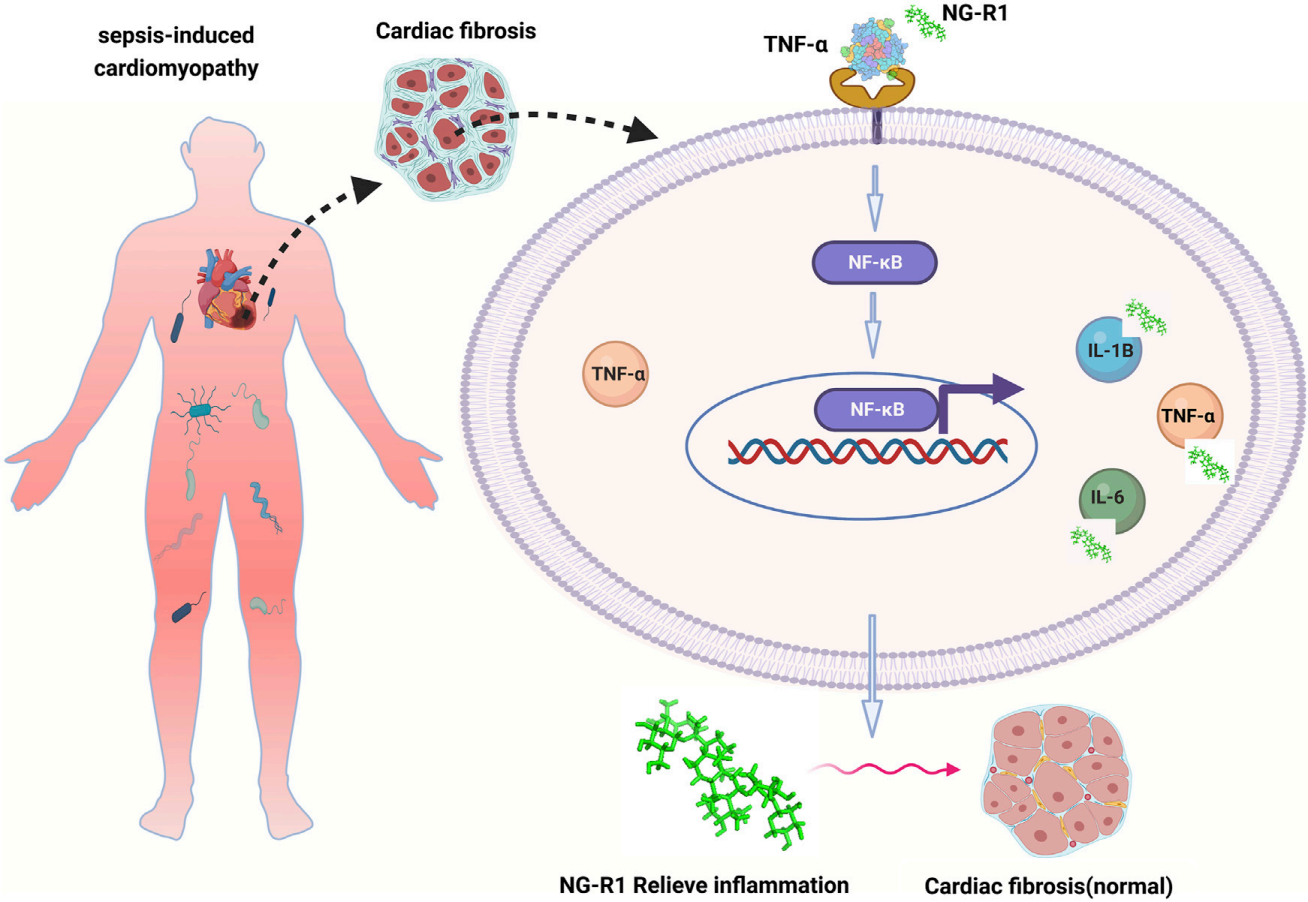
The mechanism of NG-R1 action on SIC is summarized in the figure. TNF-α is the core target of anti-inflammation in SIC, and NG-R1 may regulate the inflammatory response during myocardial injury in sepsis by regulating the expression of TNF-α, thereby reducing cardiac injury and cardiac dysfunction.

In our study, TNF-α is a core target of anti-inflammation in SIC, and NG-R1 may regulate the inflammatory response during myocardial injury in sepsis by mediating TNF-α expression, thereby alleviating cardiac injury and cardiac dysfunction (Figure 9).

## 5 Conclusion

This is the first study to investigate the mechanism of NG-R1 for the treatment of sepsis-induced myocardial injury using network pharmacology, molecular docking, and experimental validation. In summary, NG-R1 reduced myocardial injury in sepsis and inhibited the overexpression of inflammatory factors *via* network pharmacology and experimental validation. This study offers a novel approach to drug development for myocardial injury in sepsis. However, due to the general nature of network pharmacology studies, some drug or disease target genes may not be included in public databases, and the exact mechanism of NG-R1 for the treatment of SIC requires additional *in vivo* and *in vitro* experimental validation.

## Data Availability

The original contributions presented in the study are included in the article/Supplementary Material, further inquiries can be directed to the corresponding authors.
