# Supplementary material for: Exploring the molecular mechanism of notoginsenoside R1 in sepsis-induced cardiomyopathy based on network pharmacology and experiments validation
Source: Front Pharmacol. 2023 Jan 13;14:1101240. doi: 10.3389/fphar.2023.1101240 (PMC9880176; doi:10.3389/fphar.2023.1101240)
Supplement: Supplementary file 1 [file Table1.DOCX]

Supplementary Material

# Supplementary Tables

**Table 1.** 51 potential target genes details of NG-R1 therapy for SIC.

| **No.** | **Target** | **Gene symbol** |
| --- | --- | --- |
| 1 | ATPase Na+/K+ Transporting Subunit Alpha 1 | ATP1A1 |
| 2 | Caspase 3 | CASP3 |
| 3 | Interleukin 1 Beta | IL1B |
| 4 | Nuclear Factor Kappa B Subunit 1 | NFKB1 |
| 5 | Nuclear Receptor Subfamily 3 Group C Member 1 | NR3C1 |
| 6 | Tumor Necrosis Factor-Alpha | TNF |
| 7 | Vitamin D Receptor | VDR |
| 8 | Signal Transducer And Activator Of Transcription 3 | STAT3 |
| 9 | Interleukin 2 | IL2 |
| 10 | Vascular Endothelial Growth Factor A | VEGFA |
| 11 | Fibroblast Growth Factor 2 | FGF2 |
| 12 | Heparanase | HPSE |
| 13 | Heat Shock Protein 90 Alpha Family Class A Member 1 | HSP90AA1 |
| 14 | Galectin 3 | LGALS3 |
| 15 | BCL2 Like 1 | BCL2L1 |
| 16 | Hydroxysteroid 11-Beta Dehydrogenase 2 | HSD11B2 |
| 17 | Dopamine Receptor D1 | DRD1 |
| 18 | Dopamine Receptor D2 | DRD2 |
| 19 | 5-Hydroxytryptamine Receptor 2A | HTR2A |
| 20 | Cytochrome P450 Family 2 Subfamily D Member 6 | CYP2D6 |
| 21 | Histone Deacetylase 6 | HDAC6 |
| 22 | Androgen Receptor | AR |
| 23 | Matrix Metallopeptidase 9 | MMP9 |
| 24 | Matrix Metallopeptidase 8 | MMP8 |
| 25 | DNA Topoisomerase I | TOP1 |
| 26 | Integrin Subunit Alpha V | ITGAV |
| 27 | Integrin Subunit Beta 3 | ITGB3 |
| 28 | MET Proto-Oncogene, Receptor Tyrosine Kinase | MET |
| 29 | Integrin Subunit Alpha 2b | ITGA2B |
| 30 | Protein Tyrosine Phosphatase Non-Receptor Type 1 | PTPN1 |
| 31 | Major Histocompatibility Complex, Class I, A | HLA-A |
| 32 | Coagulation Factor IX | F9 |
| 33 | SRC Proto-Oncogene, Non-Receptor Tyrosine Kinase | SRC |
| 34 | Growth Factor Receptor Bound Protein 2 | GRB2 |
| 35 | Cathepsin B | CTSB |
| 36 | Adenosine A2a Receptor | ADORA2A |
| 37 | Integrin Subunit Alpha 4 | ITGA4 |
| 38 | Solute Carrier Family 37 Member 4 | SLC37A4 |
| 39 | Adrenoceptor Beta 2 | ADRB2 |
| 40 | Mitogen-Activated Protein Kinase 1 | MAPK1 |
| 41 | Purinergic Receptor P2Y12 | P2RY12 |
| 42 | Angiotensin II Receptor Type 1 | AGTR1 |
| 43 | Protein Tyrosine Phosphatase Receptor Type C | PTPRC |
| 44 | Kinase Insert Domain Receptor | KDR |
| 45 | Mechanistic Target of Rapamycin Kinase | MTOR |
| 46 | Phosphatidylinositol-4,5-Bisphosphate 3-Kinase Catalytic Subunit Gamma | PIK3CG |
| 47 | Phosphatidylinositol-4,5-Bisphosphate 3-Kinase Catalytic Subunit Alpha | PIK3CA |
| 48 | Peroxisome Proliferator Activated Receptor Gamma | PPARG |
| 49 | Peroxisome Proliferator Activated Receptor Alpha | PPARA |
| 50 | Complement C3a Receptor 1 | C3AR1 |
| 51 | 6-Phosphofructo-2-Kinase/Fructose-2,6-Biphosphatase 3 | PFKFB3 |
